# Evaluation of the Efficacy of Lusutrombopag for Chronic Liver Disease Based on Pre‐Treatment Platelet Counts: A Retrospective Multicenter Study

**DOI:** 10.1002/jgh3.70081

**Published:** 2024-12-31

**Authors:** Takayoshi Suga, Satoru Kakizaki, Atsushi Naganuma, Takeshi Hatanaka, Satoshi Takakusagi, Daichi Takizawa, Hirotaka Arai, Takashi Ueno, Keisuke Iizuka, Toru Fukuchi, Shuichi Saito, Hiroki Tojima, Yuichi Yamazaki, Toshio Uraoka

**Affiliations:** ^1^ Department of Gastroenterology NHO Shibukawa Medical Center Shibukawa Japan; ^2^ Department of Clinical Research NHO Takasaki General Medical Center Takasaki Japan; ^3^ Department of Gastroenterology NHO Takasaki General Medical Center Takasaki Japan; ^4^ Department of Gastroenterology Gunma Saiseikai Maebashi Hospital Takasaki Japan; ^5^ Department of Gastroenterology and Hepatology Kusunoki Hospital Takasaki Japan; ^6^ Department of Gastroenterology Maebashi Red Cross Hospital Takasaki Japan; ^7^ Department of Internal Medicine Isesaki Municipal Hospital Takasaki Japan; ^8^ Department of Internal Medicine Kiryu Kosei General Hospital Takasaki Japan; ^9^ Department of Gastroenterology Public Tomioka General Hospital Takasaki Japan; ^10^ Department of Gastroenterology and Hepatology Gunma University Graduate School of Medicine Takasaki Japan

**Keywords:** chronic liver disease, lusutrombopag, thrombocytopenia

## Abstract

**Background:**

Oral thrombopoietin receptor agonists are used to treat thrombocytopenia in patients with chronic liver disease who are scheduled for invasive procedures. The efficacy of lusutrombopag based on the pretreatment platelet count was investigated.

**Methods:**

Patients treated at nine hospitals from December 2015 to December 2023 were included. Efficacy was assessed by comparing the proportion of patients achieving a platelet count ≥ 50 000/μL and the change in platelet count.

**Results:**

Seventy patients were eligible for evaluation. Patients with a pretreatment platelet count < 40 000/μL had a significantly lower rate of achieving a platelet count of ≥ 50 000/μL than those with a pretreatment count of 40 000–50 000/μL (62.5% vs. 84.2%, *p* = 0.038); however, there was no significant difference in the change in platelet count (25 700 vs. 24 400/μL, *p* = 0.972). Patients with viral‐related cirrhosis showed a significantly greater change in platelet count than the others (29 100 vs. 19 200/μL, *p* = 0.012). For patients receiving multiple lusutrombopag treatments, the change in platelet count was significantly lower in the second treatment than in the first treatment (26 900 vs. 20 800/μL, *p* = 0.041). The main adverse event observed was thrombosis (2.9%).

**Discussion:**

Lusutrombopag increases platelet count regardless of pretreatment levels, but efficacy, defined as achieving a platelet count of ≥ 50 000/μL, may be insufficient in patients with a pretreatment platelet count < 40 000/μL. Additionally, patients with non‐viral liver disease responded less well to treatment compared to those with viral liver disease. Therefore, treatment strategies should be tailored based on pretreatment platelet counts and the etiology of liver disease.

## 1 Introduction

Thrombocytopenia is a common complication in patients with chronic liver disease and increases the risk of bleeding during invasive procedures [1]. Generally, a platelet count of at least 50 000/μL is desired before invasive procedures to avoid bleeding events [2, 3]. Platelet transfusion is the standard treatment for severe thrombocytopenia, but it carries various risks, including viral infection, allergic hemolytic reactions, and ABO incompatibility.

Recently, thrombopoietin receptor agonists have emerged as alternative treatments for platelet transfusions [4, 5]. Lusutrombopag and avatrombopag are thrombopoietin receptor agonists used for thrombocytopenia in patients with chronic liver disease and have demonstrated favorable efficacy and safety profiles in clinical trials [6, 7, 8, 9, 10, 11, 12, 13, 14]. The dose of avatrombopag is adjusted based on whether the baseline platelet count is below 40 000/μL, with patients receiving either 40 or 60 mg daily for 5 days [15]. Avatrombopag has been reported to exert a dose‐dependent effect on severe thrombocytopenia [16]. However, lusutrombopag does not have dose adjustments based on baseline platelet counts, and its efficacy is not well understood.

Therefore, the primary endpoint of this study was to investigate the efficacy of lusutrombopag based on whether the baseline platelet count is below 40 000/μL. The secondary and exploratory endpoints were to investigate whether the severity of chronic liver dysfunction, liver disease etiology, and the number of doses of lusutrombopag would affect its efficacy.

## 2 Patients and Methods

A retrospective multicenter study was conducted on patients with chronic liver disease treated with lusutrombopag. For each treatment, 3 mg of lusutrombopag was administered 8–14 days before the invasive procedure. Patients with chronic liver disease with platelet counts less than 50 000/μL or those deemed eligible by their attending physicians received lusutrombopag. The time course of administration of lusutrombopag is shown in Figure S1. Platelet transfusions were administered when deemed necessary for invasive procedures by the attending physician.

**FIGURE 1:**
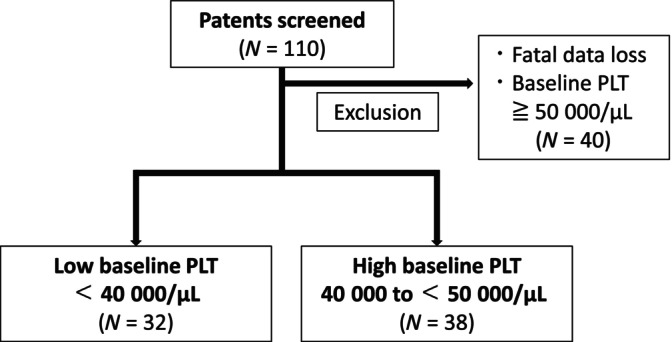
Patient screening and group classification based on baseline platelet counts (PLT). This flowchart illustrates the screening process for patients considered for inclusion in this study. Initially, 110 patients were included in the study. Exclusions were made for cases with significant data loss and baseline platelet count (PLT) ≥ 50 000/μL, totaling 40 patients. The remaining patients were categorized based on their baseline PLT: 38 patients with a higher baseline PLT (40 000 to < 50 000/μL) and 32 patients with a lower baseline PLT (< 40 000/μL).

From December 2015 to December 2023, a total of 110 patients with chronic liver disease received lusutrombopag treatment at our 9 Japanese institutions. Among these patients, we excluded those with significant data deficits or baseline platelet counts above 50 000/μL. Consequently, the remaining 70 patients were included in this study. Platelet counts were measured during the screening period and at baseline at least 1 day apart, and the mean value was used to determine eligibility. The 70 patients were divided into two groups according to their baseline platelet levels: those with < 40 000/μL, and those with between 40 000 and 50 000/μL. The pretreatment platelet count threshold was set at 40 000/μL, the same value used in previous studies [14, 15]. The patient selection process is shown in Figure 1.

### 2.1 Evaluation of Clinical Outcomes

The primary endpoint was to compare the efficacy of lusutrombopag between patients with pretreatment platelet counts of < 40 000/μL and those with platelet counts between 40 000 and 50 000/μL. Efficacy was defined as (1) the percentage of patients who did not require platelet transfusions, (2) the percentage of patients who achieved a platelet count of 50 000/μL on the day of the invasive procedure, (3) the change in platelet count from baseline to the day of the invasive procedure, and (4) the change in platelet count from baseline at each visit. The platelet count threshold for assessing treatment efficacy was set at 50 000/μL in accordance with previous similar studies [2, 3, 14, 15]. The secondary efficacy endpoints were the efficacy of lusutrombopag according to hepatic disease etiology or hepatic function. The exploratory endpoint was a comparison of the efficacy of lusutrombopag between the first and second doses in the same patients who received multiple doses. Exploratory endpoints were also evaluated in the subgroup analyses. Efficacy assessments included platelet counts at screening, baseline, day 5, procedure day, and days 7–14 post‐procedure, as well as platelet transfusion use. All adverse events were recorded regardless of whether they were related to the study drug or procedure.

### 2.2 Statistical Analysis

Categorical variables were presented as numbers (percentages) and compared using the chi‐squared or Fisher's exact test, as appropriate. Continuous variables were reported as medians [interquartile range] or means ± standard error and compared using Student's *t*‐test or the Mann–Whitney *U*‐test, as appropriate. The paired‐samples *t*‐test or Wilcoxon signed‐rank test, as appropriate, was used for paired data comparisons. *p* < 0.05 were considered statistically significant. All statistical analyses were conducted using the EZR software (Saitama Medical Center, Jichi Medical University, Saitama, Japan).

## 3 Results

### 3.1 Patient Characteristics

The patient characteristics are listed in Table 1. The median age of the patients was 70.5 [29–89] years, with 35 (50%) being male. All 70 patients were considered to have liver cirrhosis. Thirty‐six patients (51.4%) had liver function classified as Child‐Pugh A, 41 (58.6%) had hepatocellular carcinoma, and 48 (70%) had esophagogastric varices. The mean platelet count before the first lusutrombopag was 3.92 ± 0.77 × 10^4^/μL. Lusutrombopag was administered twice in 15 patients, three times in six patients, four times in four patients, and five or more times in three patients. Chronic hepatitis C was the most common etiology of liver disease (45.7%). The procedures for which lusutrombopag was administered included radiofrequency ablation (RFA), endoscopic injection sclerotherapy (EIS), and transcatheter arterial chemoembolization (TACE).

**TABLE 1:** Patient characteristics treated with lusutrombopag according to baseline platelet count.

Characteristic	Overall (*n* = 70)	< 40 000/μL (*n* = 32)	40 000 to < 50 000/μL (*n* = 38)	*p*
Mean age (±SD)	67.9 (±11.4)	68.6 (±11.3)	67.3 (±11.7)	0.66
Median age [min, max]	70.5 [29, 89]	71.0 [63.75, 76.25]	70.0 [63.0, 73.75]	0.587
< 65 years (%)	21 (30)	9 (28.1)	12 (31.6)	0.618
65 ≦ Age < 75 years (%)	31 (44.3)	13 (40.6)	18 (47.4)	
≧ 75 years (%)	18 (25.7)	10 (31.2)	8 (21.1)	
Male (%)	50	14 (43.8)	21 (55.3)	0.472
Female (%)	50	18 (56.2)	17 (44.7)	
Baseline platelet count	4.03 [0.95, 4.95]	3.50 [3.00, 3.75]	4.45 [4.19, 4.70]	< 0.001
Hepatocellular carcinoma, *n* (%)	41 (58.6)	18 (56.2)	23 (60.5)	0.809
Esophageal and gastric varices, *n* (%)	48 (70)	20 (64.5)	28 (73.7)	0.442
Child‐Turcotte‐Pugh class, *n* (%)				
Class A	36 (51.4)	13 (40.6)	23 (60.5)	0.054
Class B	31 (44.3)	16 (50.0)	15 (39.5)	
Class C	3 (4.3)	3 (9.4)	0 (0)	
ALBI score	−1.99 [−3.26, −0.75]	−1.80 [−2.24, −1.59]	−2.10 [−2.43, −1.83]	0.064
Disease etiology, *n* (%)				
Alcoholic liver disease	12 (17.2)	6 (18.8)	6 (15.8)	0.71
Chronic HBV hepatitis	4 (5.7)	1 (3.1)	3 (7.9)	
Chronic HCV hepatitis	32 (45.7)	17 (53.1)	15 (39.5)	
Metabolic steatohepatitis	14 (20)	5 (15.6)	9 (23.7)	
Other	8 (11.4)	3 (9.4)	5 (13.2)	
Antiviral therapy				
Nucleotide/nucleoside analogues for HBV	2/4 (50.0)	0/1 (0)	2/3 (66.7)	1
Direct‐acting antivirals for HCV	19/32 (59.4)	10/17 (58.8)	9/15 (60.0)	1
Scheduled procedure, *n* (%)				
Radiofrequency ablation	22 (31.4)	10 (31.3)	12 (31.6)	0.43
Endoscopic injection sclerotherapy	17 (24.3)	6 (18.8)	11 (28.9)	
Transcatheter arterial chemoembolization	11 (15.7)	4 (12.5)	7 (18.4)	
Other	20 (28.6)	12 (37.5)	8 (21.1)	

The characteristics of the patient groups, divided into those with pretreatment platelet counts of < 40 000/μL and those with counts between 40 000 and 50 000/μL, are listed in Table 1. No significant differences were observed between the two groups except for the pretreatment platelet counts.

### 3.2 Treatment Efficacy

**FIGURE 2:**
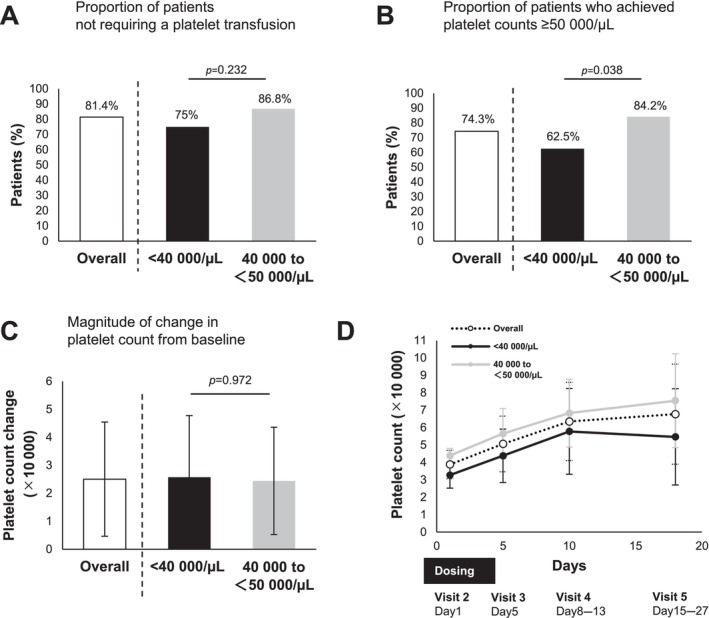
Efficacy of lusutrombopag in patients with different baseline platelet levels (PLT). (A) Proportion of patients who did not require platelet transfusion on the day of invasive procedures. (B) Proportion of patients achieving platelet counts ≥ 50 000/μL on the day of the invasive procedure. (C) Magnitude of change in platelet count from baseline on the day of the invasive procedure. (D) Platelet counts at various time points (days 1, 5, 8–13, and 15–27). Data are the mean ± SD.

Overall, 81.4% of patients treated with lusutrombopag did not require platelet transfusions (Figure 2A), 74.3% achieved a platelet count of 50 000/μL on the day of the invasive procedure (Figure 2B), and the mean absolute change in platelet count from baseline on the day of the invasive procedure was 2.50 × 10^4^/μL (Figure 2C).

### 3.3 Primary Efficacy Endpoint

The percentage of patients not requiring platelet transfusion with lusutrombopag was comparable regardless of baseline platelet count (75% vs. 86.8%, respectively; *p* = 0.232) (Figure 2A). Patients with a pretreatment platelet count < 40 000/μL had a significantly lower rate of achieving a platelet count of ≥ 50 000/μL than those with a pretreatment count of 40 000–50 000/μL (62.5% vs. 84.2%, *p* = 0.038) (Figure 2B); however, there was no significant difference in the change in platelet count (Figure 2C,D).

### 3.4 Secondary Efficacy Endpoint

**FIGURE 3:**
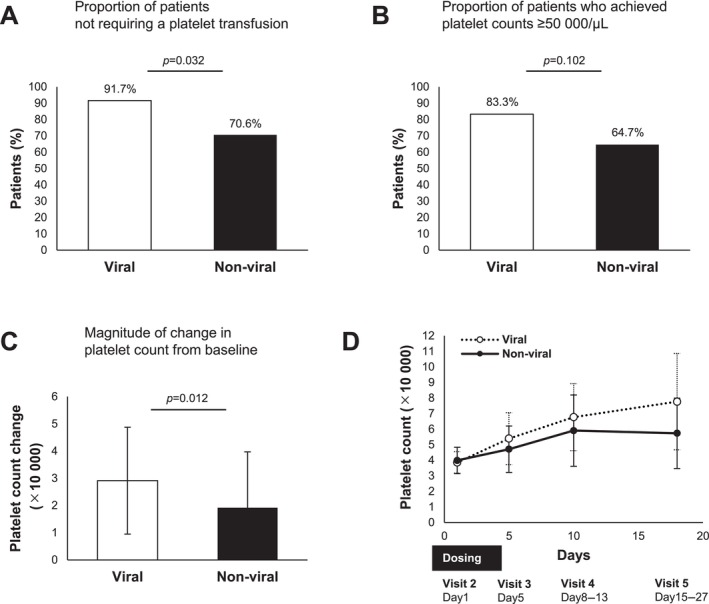
Comparative analysis of lusutrombopag efficacy in patients classified by liver disease etiology. (A) Proportion of patients who did not require platelet transfusion on the day of invasive procedures in the viral and non‐viral patient groups. (B) Proportion of patients achieving platelet counts ≥ 50 000/μL on the day of the invasive procedure in the viral and non‐viral patient groups. (C) Magnitude of change in platelet count from baseline on the day of the invasive procedure in the viral and non‐viral patient groups. (D) Platelet counts at various time points (days 1, 5, 8–13, and 15–27) in the viral and non‐viral patient groups. Data are the mean ± SD.

The characteristics of the patient groups divided into those with hepatitis B or C virus (viral) and those with other liver disease etiologies (non‐viral) are shown in Table 2. No significant differences were found between the two groups. Lusutrombopag treatment in viral patients showed a significantly higher proportion of patients not requiring platelet transfusions than in non‐viral patients (91.7% vs. 70.6%, *p* = 0.032) (Figure 3A). Lusutrombopag treatment in viral patients tended to achieve a higher proportion of platelet counts above 50 000/μL on the day of the invasive procedure than in non‐viral patients, but this difference was not significant (83.3% vs. 64.7%, *p* = 0.102) (Figure 3B). Patients with viral hepatitis had significantly greater changes in platelet levels from baseline to the day of the invasive procedure with lusutrombopag treatment than those with non‐viral etiologies (Figure 3C). The platelet counts at each visit are shown in Figure 3D. Lusutrombopag treatment in patients with Child–Pugh class A showed a significantly higher proportion of patients not requiring platelet transfusions than in those with Child–Pugh class B and C. However, the proportion of patients with platelet counts > 50 000/μL on the day of the invasive procedure and changes in platelet count from baseline were comparable between the groups (Figure S2A–D).

**TABLE 2:** Patient characteristics treated with lusutrombopag (viral or non‐viral).

Characteristic	Viral (*n* = 36)	Non‐viral (*n* = 34)	*p*
Age	68 [62.75, 73]	71 [66.25, 76]	0.264
Male, *n* (%)	19 (52.8)	16 (47.1)	0.811
Female, *n* (%)	17 (47.2)	18 (52.9)	
Baseline platelet count	3.9 [3.375, 4.15]	4.2 [3.5, 4.6]	0.255
Hepatocellular carcinoma, *n* (%)	25 (69.4)	16 (47.1)	0.089
Esophageal and gastric varices, *n* (%)	24 (66.7)	24 (72.7)	0.612
Child‐Turcotte‐Pugh score	6.0 [6.0, 7.5]	6.5 [5.25, 8.0]	0.936
Class A, *n* (%)	19 (52.8)	17 (50.0)	1
Class B or C, *n* (%)	17 (47.2)	17 (50.0)	
ALBI score	−1.91 [−2.36, −1.71]	−2.15 [−2.42, −1.73]	0.522

### 3.5 Exploratory Efficacy Endpoint

**FIGURE 4:**
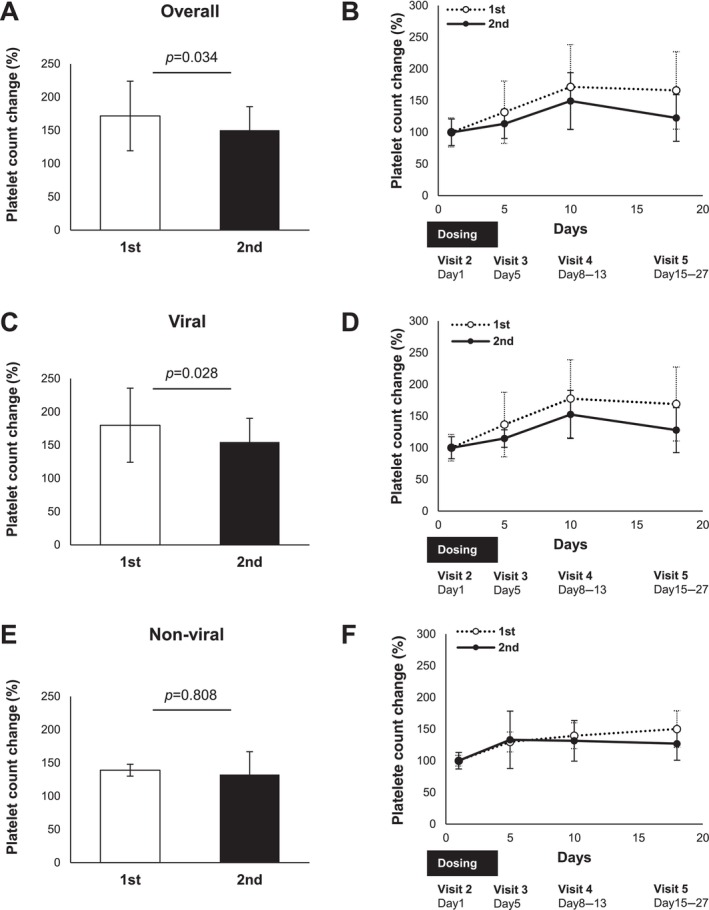
Analysis of the efficacy of multiple doses of lusutrombopag in patients with viral and non‐viral liver diseases. (A) Overall change in platelet count from baseline on the day of the invasive procedure at the first and second doses. (B) Platelet counts at various time points (days 1, 5, 8–13, and 15–27) after the first and second doses. (C) Subgroup analysis of changes in platelet count from baseline on the day of the invasive procedure with the first and second doses in patients with viral liver disease. (D) Platelet counts at various time points (days 1, 5, 8–13, and 15–27) after the first and second doses in patients with viral liver disease. (E) Subgroup analysis of changes in platelet count from baseline on the day of the invasive procedure with the first and second doses in patients with non‐viral liver disease. (F) Platelet counts at various time points (days 1, 5, 8–13, and 15–27) after the first and second doses in patients with non‐viral liver disease. Data are the mean ± SD.

Lusutrombopag treatment resulted in a significantly greater change in platelet count from baseline to the day of the invasive procedure in the same patient at the first dose than at the second dose (Figure 4A). The percentage change in platelet count at each visit from baseline is shown in Figure 4B. Subgroup analysis of the same patients receiving multiple doses of lusutrombopag showed that the change in platelet count from baseline to the day of the invasive procedure was greater with the first dose than with the second dose in patients with viral hepatitis (Figure 4C); however, this change was comparable in patients with non‐viral liver disease (Figure 4E). The percentage change in platelet count from the baseline at each visit in the subgroup analyses is shown in Figure 4D,F.

### 3.6 Adverse Effects

Adverse events recorded, whether or not related to the study drug or treatment, are shown in Table 3. Overall, there was one case of portal vein thrombosis and one case of ascites. There were no other adverse events due to lusutrombopag treatment.

**TABLE 3:** Any adverse events observed during treatment (≧ grade 3).

Adverse events, *n* (%)	Overall (*n* = 70)	< 40 000/μL (*n* = 32)	40 000 to < 50 000/μL (*n* = 38)
Dizziness	0	0	0
Eye disorder	0	0	0
Gastroenteritis	0	0	0
Abdominal pain	0	0	0
Constipation	0	0	0
Diarrhea	0	0	0
Nausea	0	0	0
Dyspepsia	0	0	0
Stomatitis	0	0	0
Fatigue	0	0	0
Pyrexia	0	0	0
Thrombosis	2 (2.9)	1 (3.1)	0
Portal vein thrombosis	1 (1.4)	0	1 (2.6)
Loss of appetite	0	0	0
Musculoskeletal pain	0	0	0
Headache	0	0	0
Edema peripheral	0	0	0
Oropharyngeal pain	0	0	0
Ascites	1 (1.4)	0	1 (2.6)

## 4 Discussion

The present study described four novel findings, including exploratory endpoints: (i) lusutrombopag increased platelet counts in patients regardless of pretreatment platelet counts; (ii) lusutrombopag was less effective in increasing platelet counts in patients with a pretreatment platelet count < 40 000/μL than in those with a pretreatment count of 40 000–50 000/μL, (iii) lusutrombopag was less effective in increasing platelet counts in patients with non‐viral liver disease than in those with viral hepatitis, and (iv) lusutrombopag was less effective in increasing platelet counts with the second dose than with the first dose in the same patient who received multiple doses of lusutrombopag.

Previous studies have reported that 79.2% of patients did not require platelet transfusions, and 77.1% achieved platelet counts ≥ 50 000/μL with lusutrombopag treatment [17]. The overall proportion of patients who did not require platelet transfusions and the proportion of patients who achieved platelet counts ≥ 50 000/μL in our study were 81.4% and 74.3%, respectively (Figure 2A,B), which was almost consistent with these results. Although spleen volume and white blood cell count are known to affect the efficacy of lusutrombopag [18, 19], whether baseline platelet count affects the efficacy of lusutrombopag is controversial. The efficacy of lusutrombopag was reported to be poor in patients with baseline platelet counts ≤ 30 000/μL [20], whereas the efficacy of lusutrombopag was reported to be equivalent in patients with platelet counts ≥ 50 000/μL and those with platelet counts < 50 000/μL [21]. The recommended dose of lusutrombopag is 3 mg once daily for 7 days prior to treatment, and unlike avatrombopag, it is not adjusted based on whether the baseline platelet count is < 40 000/μL. In the present study, lusutrombopag showed a similar increase in platelet counts in patients with baseline platelet counts of < 40 000/μL and those with platelet counts between 40 000 and 50 000/μL (Figure 2C,D). However, patients with baseline platelet counts of < 40 000/μL were less likely to achieve platelet counts ≥ 50,000/μL on the day of invasive procedures with lusutrombopag treatment (Figure 2B). Based on these results, it might be reasonable to increase the dose of avatrombopag in patients with baseline platelet counts of < 40 000/μL.

We found novel hypothetical results suggesting that the etiology of liver disease in patients with viral hepatitis and non‐viral liver disease may influence the efficacy of lusutrombopag (Figure 3A,D). This result may be explained by differences in the pathophysiological background of liver disease. It is known that hepatitis C virus viremia is associated with low platelet counts, independent of liver fibrosis [22]. In addition, patients with hepatitis B and/or C have been reported to have a higher risk of developing thrombocytopenia than those with other chronic liver diseases [23]. Patients with non‐alcoholic fatty liver disease without cirrhosis have a low prevalence of thrombocytopenia [24]. Patients with non‐alcoholic fatty liver disease were reported to have significantly higher platelet counts than those with hepatitis C virus, following stratification according to the stage of liver fibrosis [25]. Primary biliary cirrhosis patients are known to have better coagulation and less hyperfibrinolysis than other liver diseases [26]. Based on these previous reports, we hypothesized that patients with viral liver disease might have better liver function and respond better to lusutrombopag than those with non‐viral liver disease in cases of similar degrees of thrombocytopenia.

Previous studies have shown that the efficacy of lusutrombopag was consistent between the first and repeated doses [27, 28]. However, in our study, the efficacy of lusutrombopag was attenuated with the second dose compared with the first dose. This may be due to differences in the proportion of patients with viral liver disease included in the analysis; in previous reports, 64.3% (9/14) and 60% (6/10) of the patients with viral liver disease were included in the analysis [27, 28], whereas in the present study, the percentage was 80% (12/15) (Figure 4A). This consideration was consistent with the results of the subgroup analysis (Figure 4C,E).

In conclusion, lusutrombopag is effective against thrombocytopenia in patients with liver disease regardless of the baseline platelet count. However, this was a retrospective study, and the sample size was small; therefore, future prospective studies with larger patient populations are needed. The efficacy of lusutrombopag may be diminished in patients with non‐viral liver disease or with a second dose. However, this was the result of an exploratory study and requires further research to be fully elucidated.

## Ethics Statement

This study was approved by the institutional review board of NHO Takasaki General Medical Center (TGMC2023‐077) and affiliated hospitals. The study was conducted in accordance with the Declaration of Helsinki. Opt‐out consent was used in this study, and patients who did not consent to the study were excluded.

## Conflicts of Interest

The authors declare no conflicts of interest.

## Supporting information


Figure S1.

Figure S2.

## Data Availability

The data associated with the present study are available from the corresponding author upon reasonable request.
