# Supplementary material for: Evaluation of the Efficacy of Lusutrombopag for Chronic Liver Disease Based on Pre‐Treatment Platelet Counts: A Retrospective Multicenter Study
Source: JGH Open. 2024 Dec 31;9(1):e70081. doi: 10.1002/jgh3.70081 (PMC11686089; doi:10.1002/jgh3.70081)
Supplement: Supplementary file 1 — Figure S1. [file JGH3-9-e70081-s002.pptx]

## Slide 1
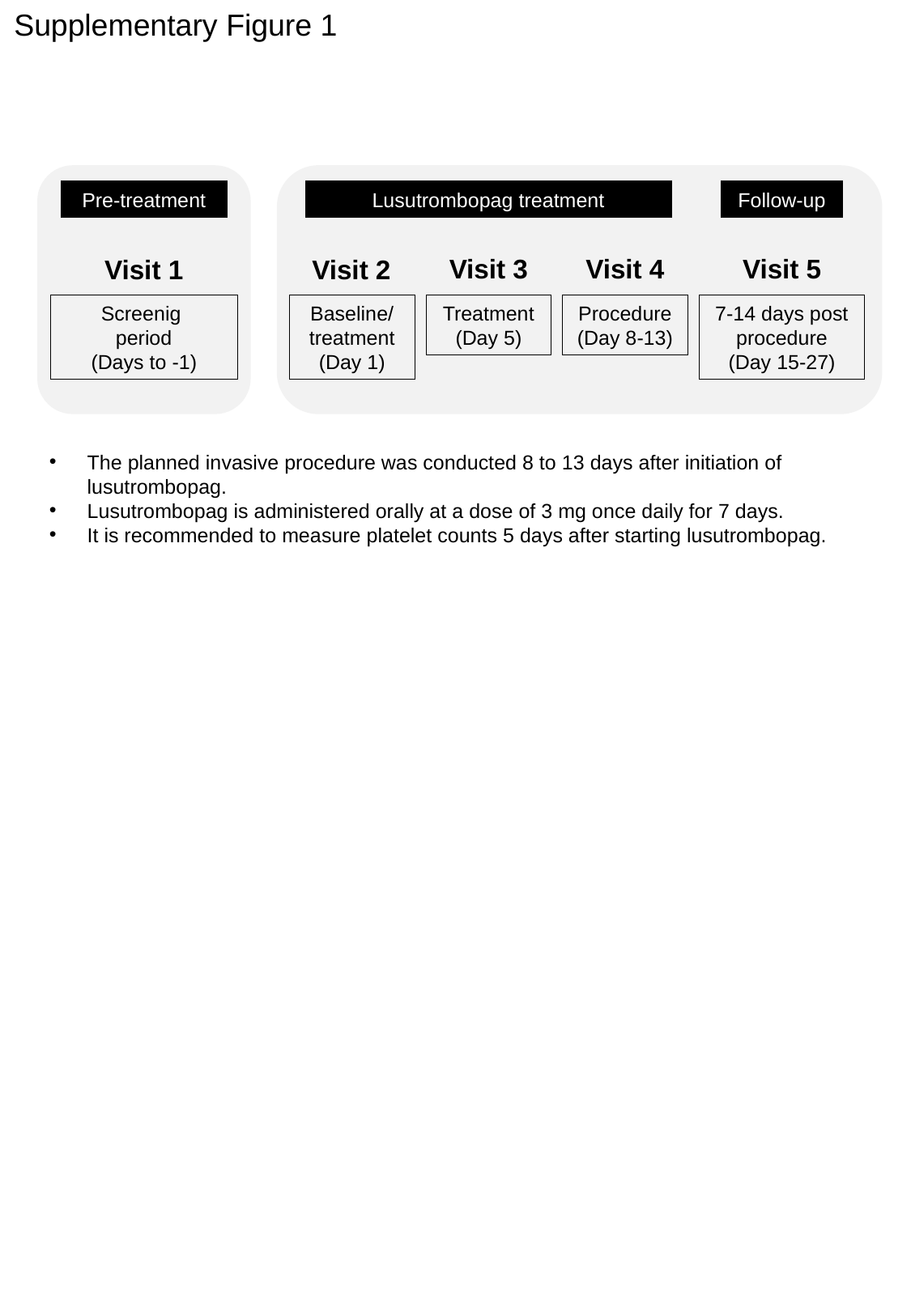

Supplementary Figure 1
Lusutrombopag treatment
Follow-up
Pre-treatment
Visit 3
Visit 4
Visit 5
Visit 1
Visit 2
Screenig
period
(Days to -1)
Baseline/
treatment
(Day 1)
Treatment
(Day 5)
Procedure
(Day 8-13)
7-14 days post
procedure
(Day 15-27)
The planned invasive procedure was conducted 8 to 13 days after initiation of lusutrombopag.
Lusutrombopag is administered orally at a dose of 3 mg once daily for 7 days.
It is recommended to measure platelet counts 5 days after starting lusutrombopag.
