# Supplementary material for: Evaluation of the Efficacy of Lusutrombopag for Chronic Liver Disease Based on Pre‐Treatment Platelet Counts: A Retrospective Multicenter Study
Source: JGH Open. 2024 Dec 31;9(1):e70081. doi: 10.1002/jgh3.70081 (PMC11686089; doi:10.1002/jgh3.70081)
Supplement: Supplementary file 2 — Figure S2. [file JGH3-9-e70081-s001.pptx]

## Slide 1
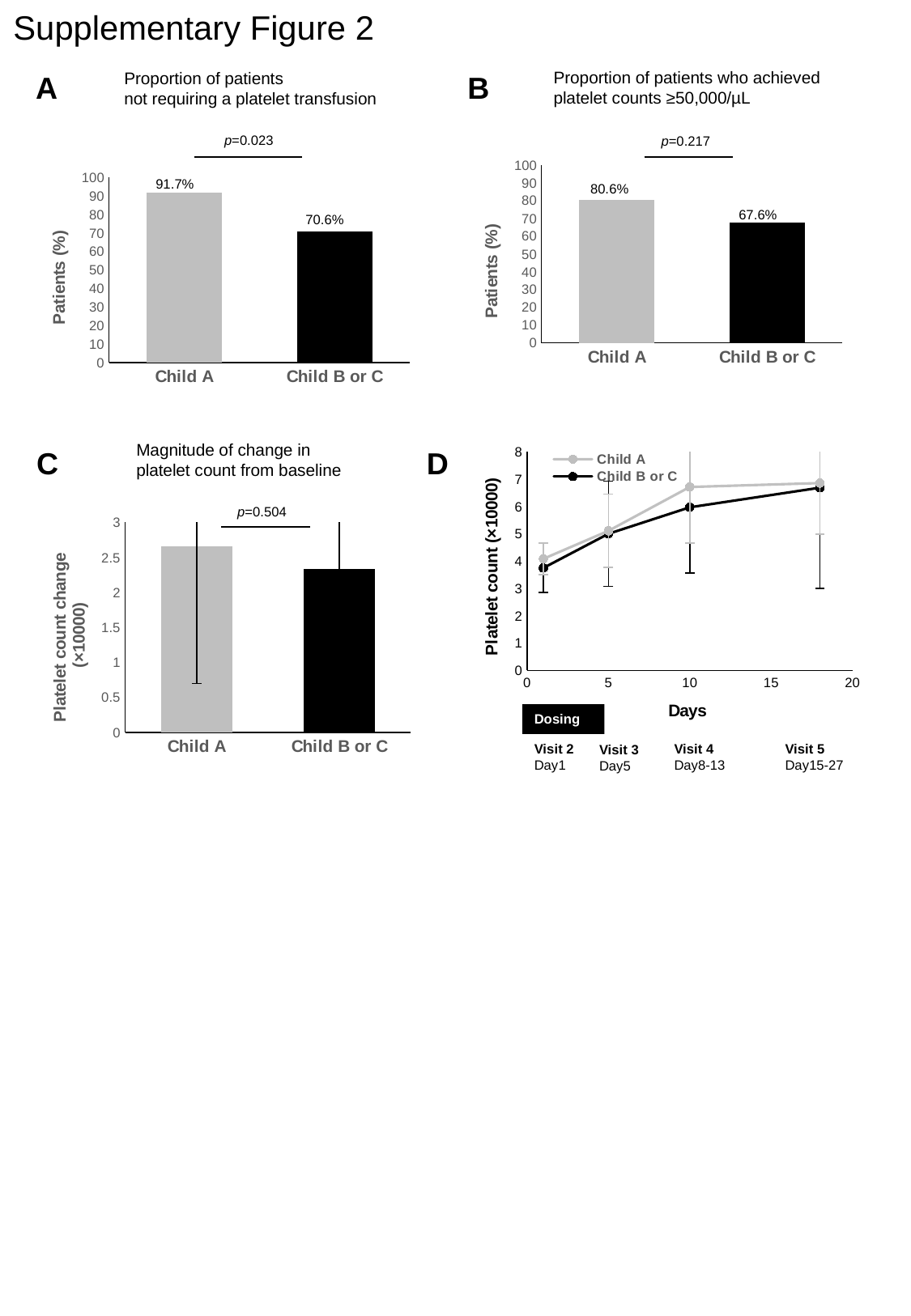

Supplementary Figure 2
Proportion of patients who achieved
platelet counts ≥50,000/µL
Proportion of patients
not requiring a platelet transfusion
A
B
p=0.023
p=0.217
### Chart
| Category | Patients % |
|---|---|
| Child A | 80.55555555555556 |
| Child B or C
 | 67.64705882352942 |80.6%
67.6%
### Chart
| Category | Patients % |
|---|---|
| Child A | 91.66666666666666 |
| Child B or C | 70.58823529411765 |91.7%
70.6%
Magnitude of change in platelet count from baseline
### Chart
| Category | Child A | Child B or C |
|---|---|---|C
D
p=0.504
### Chart
| Category | |
|---|---|
| Child A | 2.661111 |
| Child B or C | 2.332353 |Dosing
Visit 5
Day15-27
Visit 2
Day1
Visit 4
Day8-13
Visit 3
Day5
